# Risk of malignancy long after acute coronary syndrome in selected urban and rural areas and comparison with smoking risk: the ABC-7* study on Heart Disease

**DOI:** 10.1186/s40959-021-00094-y

**Published:** 2021-02-24

**Authors:** Giuseppe Berton, Heba T. Mahmoud, Rosa Palmieri, Fiorella Cavuto, Rocco Cordiano, Elisabetta Lorenzon, Francesco Bagato

**Affiliations:** 1 Department of Cardiology, Conegliano General Hospital, Via Brigata Bisagno, TV 31015 Conegliano, Italy; 2 The ABC Heart Disease Foundation-ONLUS, Conegliano, Italy; 3 Department of Internal Medicine and Cardiology, Adria General Hospital, Adria, Italy; 4 Department of Cardiology, Bassano del Grappa General Hospital, Bassano del Grappa, Italy; 5 Department of Cardiology, Feltre General Hospital, Feltre, Italy

**Keywords:** Acute coronary syndrome, Malignancy risk, Urban-Rural.

## Abstract

**Background:**

Increased cancer risk has been reported in patients with acute coronary syndrome (ACS).

**Objectives:**

To investigate geographic differences in risk malignancy long after ACS.

**Methods:**

We enrolled 586 ACS patients admitted to hospitals in three provinces in the Veneto region of Italy in this prospective study. Patient’s residency was classified into three urban and three nearby rural areas.

**Results:**

All (except for 3) patients completed the follow-up (22 years or death) and 54 % were living in rural areas. Sixteen patients had pre-existing malignancy, and 106 developed the disease during follow-up. Cancer prevalence was 17 % and 24 % (*p* = 0.05) and incidence of malignancy was 16 and 21/1000 person-years for urban and rural areas, respectively. In unadjusted logistic regression analysis, cancer risk increased from urban to rural areas (odds ratio [OR] 3.4;95 % confidence interval [CI] 1.7–7.1; *p* = 0.001), with little change from north to south provinces (OR 1.5;95 % CI 1.0-2.2; *p* = 0.06). Yet, we found a strong positive interaction between urban-rural areas and provinces (OR 2.1;95 % CI 1.2–3.5; *p* = 0.003). These results kept true in the fully adjusted model. Unadjusted Cox regression analysis revealed increasing hazards ratios (HRs) for malignancy onset from urban to rural areas (HR 3.0;95 % CI 1.5–6.2; *p* = 0.02), but not among provinces (HR 1.3;95 % CI 1.0–2.0; *p* = 0.14). Also, we found a strong positive interaction between geographic areas (HR 2.1;95 % CI 1.3–3.5; *p* = 0.002), even with a fully adjusted model.

**Conclusions:**

The results in unselected real-world patients demonstrate a significant geographic difference in malignancy risk in ACS patients, with the highest risk in the north-rural area.

## Introduction

Cardiovascular disease (CVD) and cancer are the two major causes of mortality worldwide [1, 2]. Recently, an increased risk of cancer development has been reported in patients affected by CVD, particularly acute coronary syndrome (ACS) [3–6]. Variation in cancer incidence has been observed between urban and rural areas for several decades, with higher risk of cancer in urban populations [7–9]. However, these differences diminished, and even inverted, over the past few years [10–13].

To the best of our knowledge, these differences have never been reported in specific populations, such as patients with ACS. In the present study, we investigated the possible difference in malignancy risk in six geographic areas of the Veneto region in Italy in an unselected sample of patients discharged alive after an index hospitalization with ACS and followed up for 22 years. As a comparison, we report the risk of neoplasia in smokers and non-smokers in the same sample of patients.

## Methods

### Patients

The ABC Study on Heart Disease is an ongoing prospective investigation designed to represent, as closely as possible, an unbiased population of patients with ACS (www.abcheartdiseasestudy.org/en/). The cohort includes Caucasian patients with definitive ACS, including ST-elevation myocardial infarction (STEMI), non-ST elevation myocardial infarction (NSTEMI), or unstable angina, who were admitted to the intensive care units of three general hospitals in the Veneto region of Italy between June 1995 and January 1998. The study protocol followed the Declaration of Helsinki and was approved by the hospitals’ ethics committees. The original aim of the ABC Study was to monitor these patients with regards to long-term natural history, both non-fatal and fatal events, and causes of death. Another study aim was to investigate the prognostic value of multiple baseline clinical variables. Criteria for diagnosis of ACS included clinical presentation, electrocardiogram findings, and the presence of serum biochemical markers of necrosis [14, 15].

**Fig. 1:**
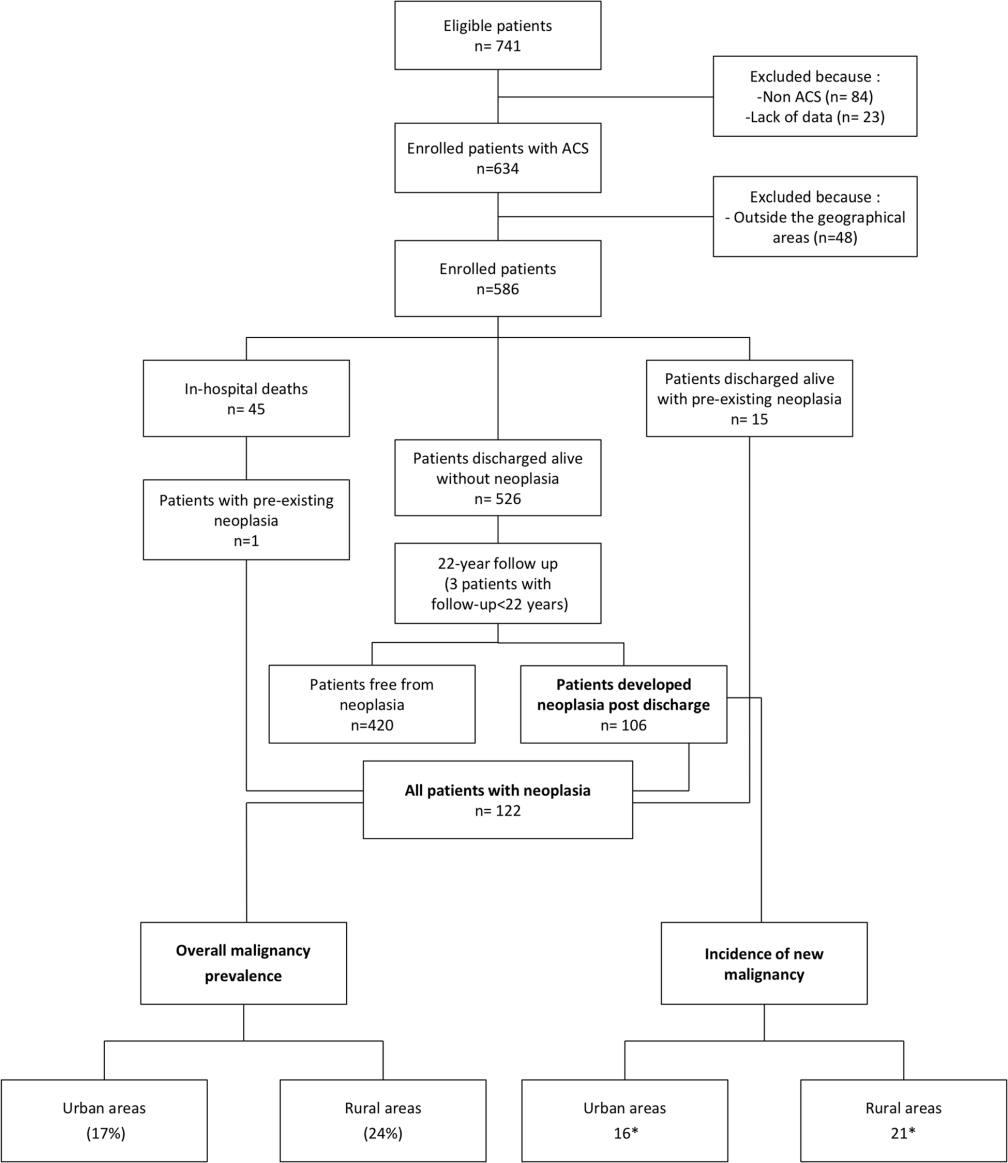
Flow diagram of the study population and progress during follow-up *Per 1000-person years ACS = acute coronary syndrome

A total of 741 patients were considered eligible upon admission, but 84 patients were excluded because they had diseases other than ACS, 23 were excluded due to a lack of baseline data, and 48 patients were not included in the study because they lived outside the Veneto region. Among the 586 patients with ACS who were enrolled in the present study, 45 died during the index hospitalization. Therefore, the post-discharge follow-up study included 541 patients (Fig. 1). Each patient received an anonymous code, and no personal data or identifiers were included in the baseline or follow-up database.

### Veneto region and urban‐rural classification

**Fig. 2:**
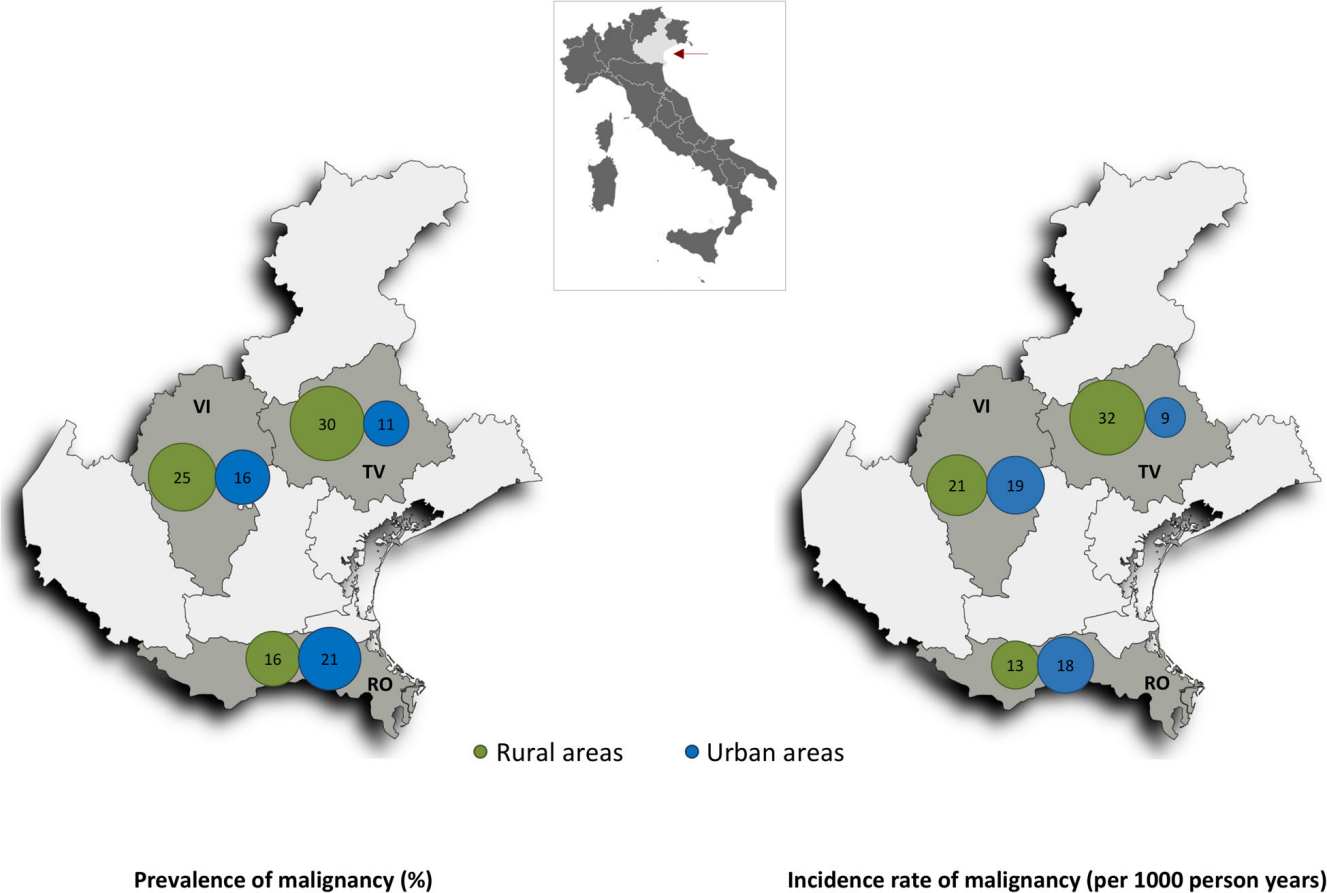
Map of the Veneto region (red arrow) and the location of the six geographic areas. The respective malignancy prevalence and incidence rates are shown in each area RV = Rovigo province; TV = Treviso province; VI = Vicenza province

Veneto is a north-eastern Italian region extending from the Alps to the Adriatic Sea (Fig. 2). The region is divided into seven provinces, and Venice is the regional capital. The population is approximately 5 million, ranking 5th in Italy. The region covers 18 399 km^2^, 6 % of the total Italian territory and going south to north it turns from flat to hilly and mountainous. The present study included patients from hospitals in Conegliano-Vittorio Veneto, Bassano, and Adria-Cavarzere, cities located in the provinces of Treviso (north), Vicenza (middle), and Rovigo (south), respectively. Urban and rural areas were designated for each province, and a residence code, that follows the national strategic Rural Development Plan (Programma di Sviluppo Rurale [PSR] 2014–2020), was assigned to each patient based on their residential address [16]. Thus, areas of residency were classified into three urban and three nearby rural areas (Fig. 2). The total population of these six areas is 586 976 inhabitants, with 24 % living in urban areas and 76 % living in rural areas [16, 17].

In July 2019, at the 43rd session of the UNESCO World Heritage Committee, “Prosecco Hills of Conegliano and Valdobbiadene” was added to the UNESCO World Heritage list as a cultural landscape [18]. This area is in Treviso province, and most of the UNESCO areas overlap the geographic area of the ABC Study.

### Measurements and follow‐up

All enrolled patients provided written informed consent. At enrolment, a thorough patient history was collected from medical records and patient interviews. All of the analysed baseline clinical and laboratory data were obtained during the first 7 days of hospitalization in the intensive coronary care unit. The ACS diagnosis criteria were the presence of at least two of the following: central chest pain lasting > 30 minutes; typical changes in serum enzymes, including total creatine kinase (CK) and creatine kinase MB (CK-MB); and typical electrocardiogram changes with pathological Q waves and/or localized ST-T changes in at least two contiguous leads [19]. Details regarding the measured variables were published previously [14, 15].

Each patient underwent a clinical check-up 1, 3, 5, 7, 10, 12, 15, 17, 20, and 22 years after recruitment. At each recruitment hospital, two cardiologists were responsible for monitoring the cohort of patients throughout the follow-up. Data were obtained from scheduled examinations, public administrations, hospital records, family doctors, post-mortem examinations, and death certificates. The medications received during the index hospitalization and follow-up treatments were also recorded. For the present study, the presence of neoplastic disease at the index admission and the incidence of new malignancy (i.e., the first documented clinical diagnosis of the disease) were recorded. All data after enrolment were recorded prospectively following the protocol of the ABC Study on Heart Disease [14]. Baseline data and follow-up data were recorded in two different datasheets. For the present analysis, the datasheets were merged after the completion of 22 years of follow-up.

### Statistical analysis

The accrued variables were analysed as continuous variables or proportions. We applied log transformations to correct for positively skewed distributions as appropriate. Measured variables were analysed using the unpaired Student’s t-test, and categorical variables using Pearson’s chi-squared test. If a patient dropped out before 22 years of follow-up, their data were censored at that time.

We constructed survival curves using cumulative hazards [20]. As a first step, the homogeneity of malignancy risks in the six geographic areas and smokers was tested using the Breslow-Day test of homogeneity, with p < 0.05 indicating dis-homogeneity of the odds ratios (ORs). To study the effect modification, a formal interaction term was assessed for overall malignancy risk between the six geographic areas in unadjusted and fully adjusted logistic regressions. Finally, we used Cox survival regressions including a formal interaction between all areas, and adjusting for the main clinical variables, to estimate the risk of neoplasia onset. Schoenfeld residuals were used to test the proportionality assumption with 95 % confidence intervals (CIs). We quantified the risk estimate as the hazard ratio (HR). To graphically show the probability of malignancy and the urban-rural HR for neoplasia onset across the north, middle, and south provinces, we utilized the marginal analysis. We assessed the risks of malignancy prevalence and neoplasia onset in smokers using the same steps for comparison.

Baseline characteristics were summarized as medians and interquartile ranges for continuous variables and numbers and percentages for categorical variables. Unless otherwise indicated, two-tailed P-values < 0.05 were considered significant. All statistical analyses were performed using STATA 14 (College Station, Texas, USA).

## Results

### Study population and baseline characteristics

Of the 586 enrolled patients, 54 % were living in rural areas of the three provinces. Table 1 summarizes the main clinical characteristics by area of residency. Both groups shared most of the clinical characteristics (Table 1). All enrolled patients completed the follow-up unless pre-empted by death, except three patients for whom survival was censored before 22 years (two withdrew consent and one moved overseas). At the time of enrolment, 16 patients had previously diagnosed malignancy; one of them died during hospitalization. A total of 106 patients developed the disease during follow–up (Fig. 1). The most frequent sites of malignancy were lung (22 %), colorectal (19 %), prostate (15 %), breast (5 %), pancreas (5 %), and leukaemia (5 %).

**Table 1:** Baseline characteristics of patients with acute coronary syndrome according to area of residency

Variable	Overall sample (*n* = 586)	Urban areas (*n* = 268 − 46 %)	Rural areas (*n* = 318 − 54 %)	*P*-value
Age, years	67(60–75)	68(59–76)	65(60–75)	0.25
Gender, female	31	30	31	0.75
Education above primary school	24	32	17	< 0.0001
Body mass index, kg/m^2^	25(23–28)	27(23–27)	25(24–28)	0.05
Smoking habit ^a^	66	66	65	0.75
Alcohol use	75	76	75	0.81
Hypertension	50	52	47	0.23
Diabetes mellitus	24	26	22	0.27
Systolic blood pressure, mmHg	120(110–130)	120(110–130)	120(110–135)	0.50
Diastolic blood pressure, mmHg	80(70–80)	75(70–80)	80(70–80)	0.53
Heart rate, beats/min	72(60–83)	73(62–82)	71(60–83)	0.82
Non-ST elevation ACS	37	36	38	0.54
Killip class > 1	37	41	34	0.10
Hb, g/dL	14(12–15)	14(12–15)	14(12–15)	0.83
Blood glucose level, mg/dL	122(101–166)	125(104–175)	119(99–156)	0.20
Serum creatinine level, mg/dL	0.9(0.9–1.1)	0.9(0.8–1.2)	0.9(0.9–1.1)	0.59
CK-MB peak^*^, U/L	103(44–212)	104(47–213)	102(43–207)	0.67
Total cholesterol^*^, mg/dL	206(175–242)	203(171–236)	208(177–243)	0.26

The values are presented as median (interquartile range) or percentages

ACS = acute coronary syndrome; CK-MB = creatine kinase-MB isoenzyme; Hb = haemoglobin

^a^Previous smokers and currently smoking patients

^*^P-values were calculated using log-transformed data

### Overall malignancy prevalence by geographic area

By the end of follow-up, the overall prevalence of neoplasia was 21 %. The prevalence was 17 % and 24 % for the urban and rural areas, respectively (*p* = 0.05; Fig. 2). The Breslow-Day test of homogeneity demonstrated a significant difference in malignancy prevalence between urban and rural areas across the three provinces (north: OR 3.4, middle: OR 1.6, and south: OR 0.7; *p* = 0.01). However, a significant difference was also observed going from south to north in each geographic area (urban: OR 0.5, rural: OR 1.9; *p* = 0.005). We found no difference across smoking habit (urban: OR 2.3, rural: OR 2.6; *p* = 0.80 and north: OR 2.0, middle: OR 5.8, south: OR 1.9; *p* = 0.21).

**Fig. 3:**
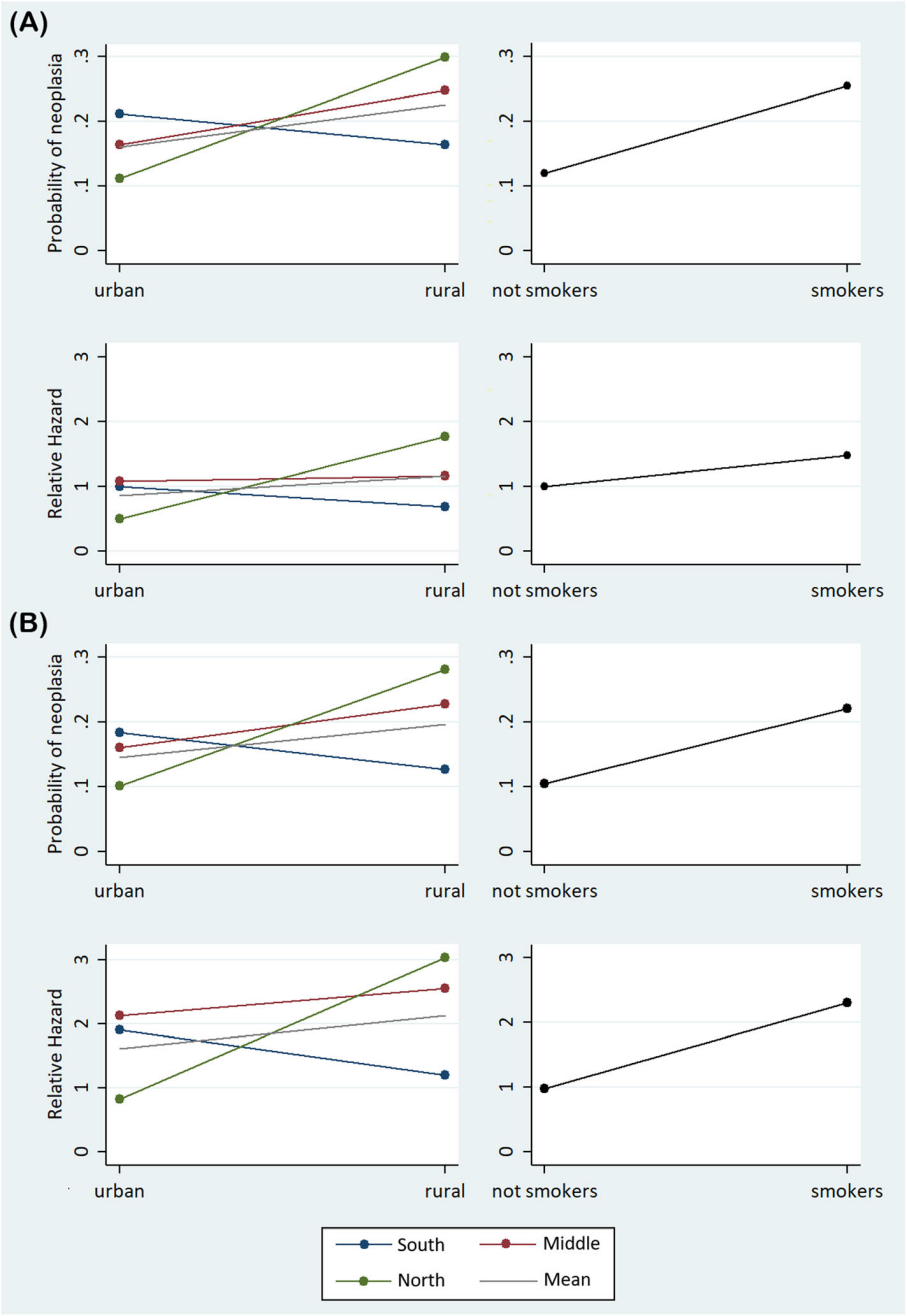
Graphical representation of the interaction analysis The probability of malignancy and the hazard ratios for neoplasia onset in urban-rural areas across three provinces are shown for patients followed 22 years after ACS. **a** Unadjusted models. **b** Fully adjusted models (for age, gender, smoking, alcohol consumption, education level, baseline serum cholesterol, and presence of heart failure at admission). The risk of neoplasia onset by smoking status is also shown for comparison

In the unadjusted logistic regression analysis (Table 2), we observed that the OR for malignancy increases from urban to rural areas, whereas only a slight change in risk was observed from north to south provinces. When a formal interaction term was considered (Table 2), we observed a strong positive interaction between the six geographic areas of residency; the risk increased from urban to rural areas when going from south to north provinces. The interaction analysis showed that the risk of malignancy was 21 % vs. 16 % in the south, 16 % vs. 25 % in the middle, and 11 % vs. 30 % in the north for urban and rural areas, respectively (Table 2; Fig. 3a). Similar results were obtained using a fully adjusted logistic model, adjusted for age, gender, smoking, alcohol consumption, education level, baseline serum cholesterol, and presence of heart failure at admission (Table 2; Fig. 3b). Table 2 also reports the cancer risk by smoking habit in both logistic regression models.

**Table 2:** Logistic regression analysis of overall malignancy risk and COX regression analysis of the risk of neoplasia onset over 22 years of follow-up after ACS with the interaction for risks between six geographic areas and by smoking habit

	Unadjusted model			Fully adjusted model		
**Logistic regression analysis (n = 586)**	**OR (95 % CI)**		***P*-value**	**OR (95 % CI)**		***P*-value**
Urban-rural areas	3.4 (1.7–7.1)		0.001	3.4 (1.6–7.3)		0.001*
North-south provinces	1.5 (1.0-2.2)		0.06	1.4 (0.9–2.1)		0.11*
Interaction (urban/rural areas and south to north provinces)	2.1 (1.2–3.6)		0.003	2.1 (1.2–3.6)		0.003*
Smoking habit	2.5 (1.6–4.1)		< 0.0001	2.4 (1.3–4.6)		0.006†
**Cox regression analysis (n = 526)**	**HR (95 % CI)**	**Z**	***P*-value**	**HR (95 % CI)**	**Z**	***P*-value**
Urban-rural areas	3.0 (1.5–6.2)	3.1	0.002	3.2 (1.6–6.6)	3.2	0.001*
North-south provinces	1.3 (1.0–2.0)	1.5	0.14	1.4 (1.0-2.1)	1.8	0.08*
Interaction (urban/rural areas and south to north provinces)	2.1 (1.3–3.5)	3.1	0.002	2.2 (1.4–3.6)	3.2	0.001*
Smoking habit	1.8 (0.8-4.0)	1.5	0.13	2.9 (1.2–7.21)	2.4	0.01†

*OR* odds ratio; *CI *confidence interval; *HR* hazard ratio

*Adjusted for age, gender, smoking, alcohol consumption, education level, baseline serum cholesterol, and presence of heart failure at admission

† Adjusted for age, gender, province, alcohol consumption, education level, baseline serum cholesterol, and presence of heart failure at admission

### Malignancy incidence rate by geographic area

The patients discharged alive without neoplasia (*n* = 526) represented 5698 person-years of follow-up. The incidence rate of new malignancy was lower in urban than rural areas (16 and 21/1000 person-years, respectively; Fig. 1). The Breslow-Day test of homogeneity revealed significant differences in the risk of malignancy onset between urban and rural areas across the three provinces (north: OR 3.8, middle: OR 1.4, and south: OR 0.8; *p* = 0.02). A significant difference was also observed from south to north in each geographic area (urban: OR 0.6, rural: OR 1.8; *p* = 0.01). We found no difference across smoking habit (urban: OR 2.3, rural: OR 2.5; *p* = 0.85 and north: OR 2.0, middle: OR 5.1, south: OR 1.9; *p* = 0.31).

**Fig. 4:**
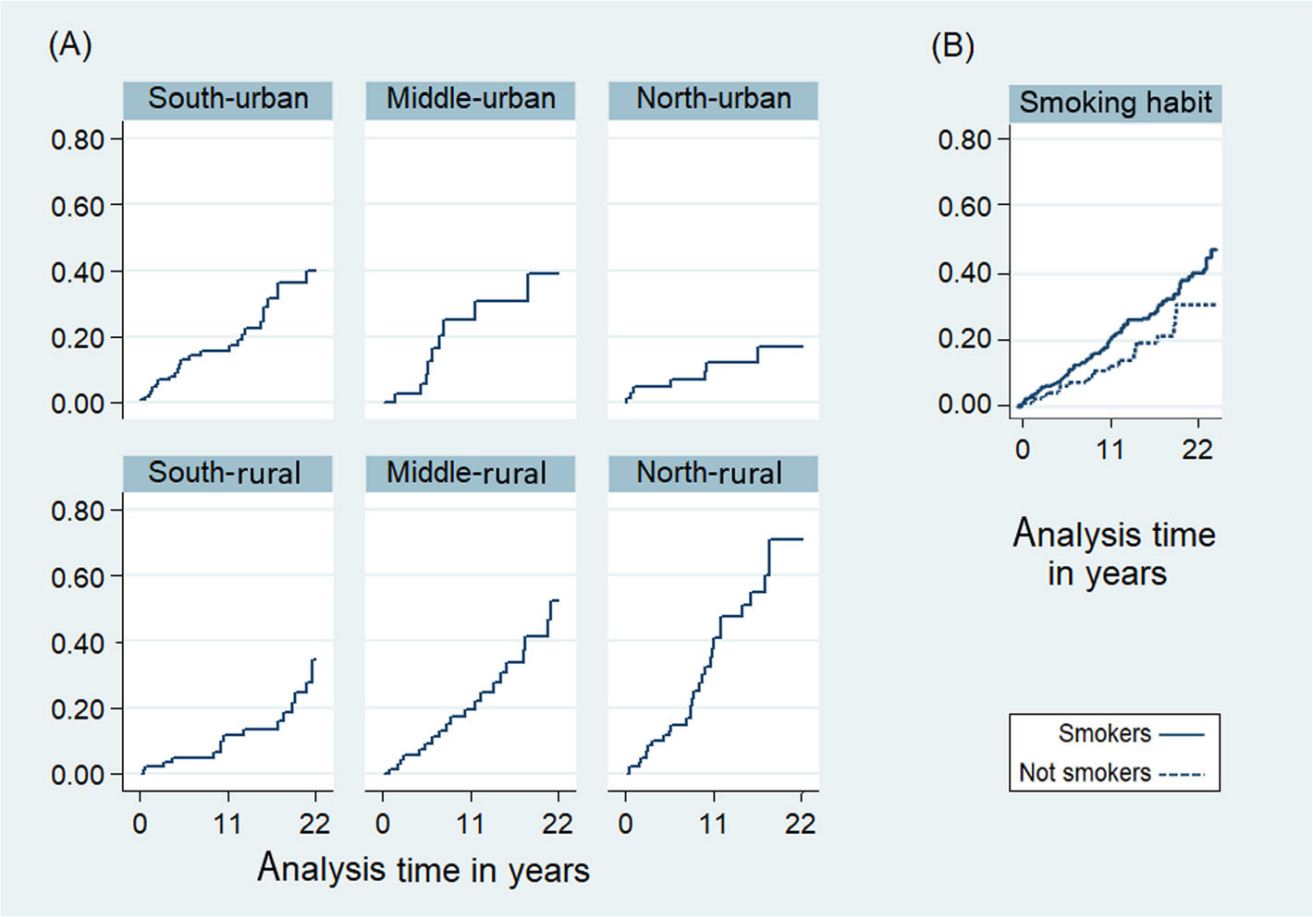
Nelson-Aalen cumulative hazard estimates for cancer risk over 22 years of follow-up by geographic area (**a**) and smoking habits (**b**)

The Nelson-Aalen cumulative hazards estimate for cancer risk across geographic areas indicated that the cumulative hazard appears to decline from south to north areas for urban inhabitants, and increases from north to south for rural inhabitants, with the highest risk in the north-rural area. The cumulative hazard was also higher among smokers than non-smokers (Fig. 4).

In the unadjusted Cox regression analysis (Table 2), we observed that the HR for malignancy onset increases from urban to rural areas, whereas no change was observed from north to south. When an interaction term was considered, we observed the same strong positive interaction between geographic areas, as the risk increased from urban to rural areas across the south to north provinces. Cox regression-based marginal analysis showed that the risk of malignancy onset was 7 vs. 29 in the south, 9 vs. 20 in the middle, and 25 vs. 16 in the north for urban and rural areas, respectively (Fig. 3a). Similar results were obtained using a fully adjusted cox regression model, adjusted for age, gender, smoking, alcohol consumption, education level, baseline serum cholesterol, and presence of heart failure at admission (Table 2; Fig. 3b). Table 2 also reports the HR for malignancy onset after ACS by smoking habit in both Cox regression models.

## Discussion

The main result of the ABC-7* Study on ACS is that urban-rural geographic areas have different long-term risk of malignancy, regarding the lack of difference in clinical and most of demographic characteristics between patients from rural and urban areas and even after controlling the survival models for age, gender, education level and main clinical features. This difference supports the hypothesis that the urban-rural geographic area is a strong independent effect modifier of malignancy risk in ACS patients.

Living in a rural area positively influences the probability of having cancer for patients in the northern province, meaning that those who live in the rural areas of this province have a greater risk of incurring neoplasia compared to citizens who live in the urban areas. In contrast, living in a rural area negatively affects the probability of having cancer in the southern province.

Notably, results from the present study also indicate that cancer risk in the north-rural area is even higher than the risk associated with tobacco smoking (Fig. 4), which is considered a leading preventable cause of cancer and cancer deaths [21, 22], and strong evidences indicate that smoking significantly increases the risk of several malignancies [23–25]. In a recent meta-analysis of 19 population-based prospective cohort studies included data for 897 021 European and American adults, current-smokers had a significantly higher overall risk of developing and dying from cancer (HR 1.44, 95 % CI 1.28–1.63 and HR 2.19, 95 % CI 1.83–2.63, respectively; P < 0.05, I^2^ > 75 %) compared to never-smokers [26], which is in concordance with our results based on smoking habits.

Urban-rural variation in cancer risk has been analyzed for several years to emphasize lifestyle dissimilarities, such as smoking and dietary habits, socioeconomic status, and exposures to other risk factors [7, 27, 28]. Yet, homogeneous exposure to the most common risk factors in both urban and rural areas, particularly in developed Western European countries, has recently been reported to be a consequence of economic development, growing homogenization of lifestyle, and increased relocation opportunities [27, 29]. Thus, several reports suggested that the differences in malignancy risk, as well as exposure to different risk factors seem to depend on health service accessibility, either diagnostic or therapeutic, and secondary prevention strategies, such as national screening programmes [27, 30, 31]. Several reports have also documented an elevated cancer risk in the general population in rural communities compared to urban areas [10–12, 30].

We also recently documented a higher long-term risk of malignancy in patients with ACS compared to the general population [4]. In our patients, the incidence of cancer was similar in men and women, and higher in older patients than in younger patients [4].

To the best of our knowledge, the present study is the first to report on the geographic distribution of malignancy in this specific population, ACS patients, with a very long follow-up and very few dropouts. In agreement with the medical knowledge, we found an association between malignancy risk and other important variables, such as age and smoking, but we also found an inverse association between malignancy risk in both urban and rural areas and serum cholesterol as in previous reports [32, 33].

The explanation for the higher risk in the north-rural area in our cohort of patients, which resulted in approximately 3-times higher risk than in the north urban area, is not yet clear as both urban and rural areas share most of the clinical and of demographic characteristics. The level of higher education was lower in rural areas (Table 1), and it has been reported that education level inversely associated with cancer incidence [34, 35]. Yet, adjustment for education level did not eliminates the risk differences of malignancy onset across the six geographic areas.

The six geographic areas considered in this analysis share the same national and regional primary healthcare services which are delivered by Health Districts, the operative branches of Local Health Units. Each Health District is responsible for planning and delivering health and social care based on population needs where a maximum of 1500 patients are assigned for each general practitioner. Moreover, recently the Veneto Region adopted a new primary care model, the Integrated Medical Group, where four or more general practitioners work together as well as with specialists, nurses and other health professionals and social workers to deliver a comprehensive array of people-centred services; ensure the effective management and care for chronic patients and take responsibility for community health [36, 37]. Thus, it seems unlikely that differences in health system organization can justify the diverse risk of malignancy reported in the present paper. Although recent reports have suggested that the gradual convergence of environmental and economic factors and the changed lifestyle may have contributed to the observed difference in cancer risk, rural residents tend to be older, have a lower income, and less education than urban residents [29, 38]. Gandinia et al. recently analysed data from 74,989 individuals aged > 35 years and living in 1442 Italian municipalities, who were recruited in 1999–2000 and followed up until 2008. In agreement with our results, they reported that the majority were living in rural areas with lower education level, and that the events of first hospital admission due to cardiovascular diseases or neoplasia were higher in rural residents [39].

Notably, the north-rural area of the present study includes 86 % of the population living in the rural areas of the Prosecco Hills of Conegliano and Valdobbiadene (UNESCO cultural landscapes), whereas the north-urban area includes 100 % of the urban UNESCO population. On the other hand, these rural UNESCO areas overlap 78 % of the population of the north-rural area of the ABC 7* Study. This observation sheds light on the higher risk of malignancy in the UNESCO areas than the other geographic areas in the present study.

One of the biggest strengths of this study is derived from the long duration of follow-up with almost no dropouts. To the best of our knowledge, no previous studies have reported such a contrast in malignancy risk between urban and rural areas for a specific population.

## Conclusions

This prospective study of unselected real-world patients demonstrated a significant difference in the overall malignancy risk, as well as neoplasia onset in different geographic areas of the Veneto region, with the highest risk in the north-rural area and the lowest risk in the north-urban area, among lifelong ACS patients. The risk seems to be higher than that observed with smoking habits. Future studies investigating the causal relationships leading to a higher risk of malignancy in certain areas and the rule of other environmental risk factors in such a geographic difference should be considered.

### Study limitations

This study also has limitations. A major limitation of the ABC Study was that, at the time of patient enrolment, percutaneous coronary angioplasty was not yet used to reopen coronary arteries in patients with STEMI. Thus, whether the results may have been altered by early mechanical reperfusion remains uncertain. Another limitation of the study is that the diagnosis of myocardial infarction did not account for troponin measurement, as it was not in use at that time; therefore, we used the rise and gradual decline of creatine kinase and creatine kinase-MB as biochemical markers of necrosis. Nevertheless, these markers of necrosis are still recommended in the absence of troponin measurement [40]. Furthermore, the absence of information on some individual risk factors as well as environmental exposures related to cancer development is also a limitation, and it would have been interesting to have another control group to establish whether incidence of malignancy is increased in that geographical area independently of the presence of acute coronary syndrome or smoking history. Also, information on the evolution of cardiovascular risk factors over the follow-up period, which undoubtedly influence the prevalence of cancer, were not available at the time of this analysis. Finally, all enrolled patients in this study were Caucasians, and we cannot generalize the present findings to regions outside north Italy or other populations and ethnic groups.

## Data Availability

The datasets used and/or analysed during the current study are available from the corresponding author on reasonable request.

## Abbreviations

CVD: Cardiovascular disease.
ACS: Acute coronary syndrome
STEMI: ST-elevation myocardial infarction
PSR: Programma di Sviluppo Rurale (Rural Development Plan)
ABC: An acronym for Adria, Bassano, Conegliano, and Padova Hospitals
CK: Creatine kinase
ORs: Odds ratio
CIs: Confidence intervals
HR: Hazard ratio
